# Unexpected remission of Darier disease in a melanoma patient treated with trametinib and naporafenib

**DOI:** 10.1016/j.jdcr.2026.05.025

**Published:** 2026-05-15

**Authors:** Karama Sboui, Nicolas Poulalhon, Luc Thomas, Stéphane Dalle

**Affiliations:** Dermatology Department, Lyon Sud Hospital, Pierre Bénite, France

**Keywords:** Darier disease, MEK inhibition, melanoma, trametinib

## Introduction

Darier disease (DD) is an inherited disorder of keratinization caused by mutations in the ATP2A2 gene, which encodes the sarco-endoplasmic reticulum calcium ATPase type 2 (SERCA2), leading to impaired keratinocyte adhesion.^1^^,^^2^ Treatment options are limited, and refractory cases remain particularly difficult to manage. Recently, Soto-García et al reported a remarkable response of severe DD to trametinib.^3^ We report an independent case of long-standing DD that showed unexpected remission during treatment with trametinib and naporafenib for metastatic melanoma, suggesting that modulation of the mitogen-activated protein kinase/extracellular signal-regulated kinase (MAPK/ERK) pathway may restore epidermal integrity.

## Case report

**Fig 1:**
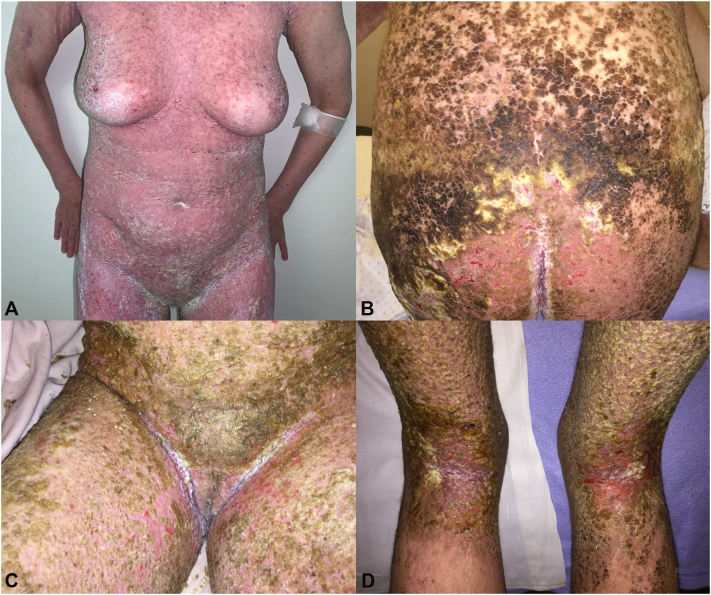
**A-D,** Severe, widespread Darier disease before initiation of combined trametinib (1 mg/day) and naporafenib (100 mg twice daily).

**Fig 2:**
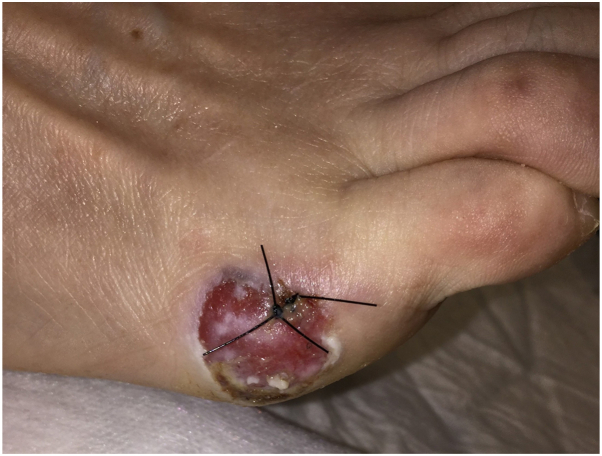
Preoperative clinical image of the acral lentiginous melanoma of the right foot.

**Fig 3:**
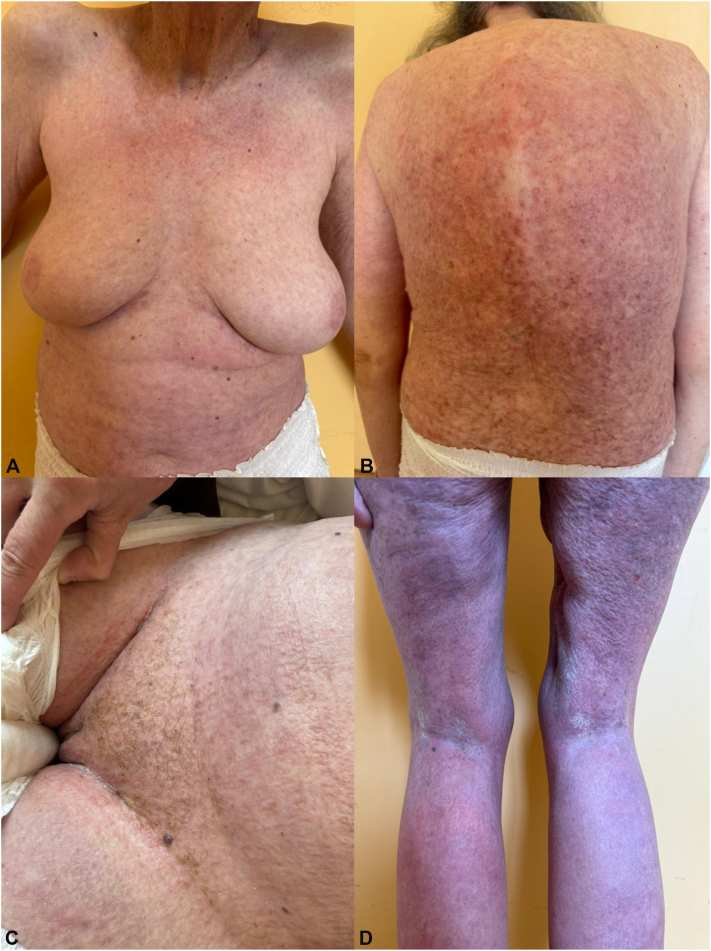
**A-D,** Same anatomical areas photographed approximately 10 months after treatment initiation, showing almost complete clearing of keratotic and erosive lesions with restoration of normal skin surface. Residual erythema and post-inflammatory hyperpigmentation can be seen on the back of the patient **(B)**.

A 64-year-old woman had a history of severe DD since adolescence, characterized by widespread keratotic and erosive lesions with recurrent bacterial, viral, and fungal infections, and numerous hospitalizations for serious flare-ups (Fig 1). Multiple therapeutic approaches, including systemic and topical retinoids, photodynamic therapy, and laser treatments had been ineffective. In 2020, she was diagnosed with an acral lentiginous melanoma (pT1bN1cM0, stage IIIB, AJCC eighth edition) harboring an NRAS Q61H mutation on her right foot (Fig 2). Following several years of surgical management and adjuvant anti-PD-1 therapy, she experienced disease progression and, in January 2025, was enrolled in the SEACRAFT-2 clinical trial (NCT06346067) evaluating combined naporafenib (100 mg twice daily) and trametinib (1 mg daily). Within 3 weeks of treatment initiation, partial clearing of the hyperkeratotic and crusted DD lesions was observed, progressing to near-complete remission by the third month (Fig 3). The improvement persisted after discontinuation of naporafenib following trial withdrawal for melanoma progression in August 2025, while the patient continued trametinib and nivolumab, with no recurrence of DD at the last follow-up in December 2025. A transient grade 3 hepatotoxicity with cytolysis and cholestasis developed shortly after treatment initiation, prompting temporary trametinib dose reduction to 0.5 mg daily, with subsequent normalization of liver function tests.

## Discussion

Experimental data have shown that loss of ATP2A2 function in keratinocytes leads to hyperactivation of the MAPK/ERK pathway and impaired desmosomal adhesion, both reversible by mitogen-activated extracellular signal-regulated kinase (MEK) inhibition.^4^ Cutaneous eruptions with features of acantholytic dyskeratosis resembling Grover or DD have been reported in patients treated with selective BRAF inhibitors, an effect attributed to paradoxical MAPK hyperactivation in normal keratinocytes leading to decreased SERCA2 expression.^5^ These drug-induced eruptions have been shown to improve with MEK inhibition.^4^ Our patient’s long-standing, treatment-refractory condition entered sustained remission shortly after initiation of combined trametinib and naporafenib for NRAS-mutant melanoma. The durable response and its persistence after naporafenib discontinuation suggest that MEK inhibition was primarily responsible for the therapeutic effect, in line with the recent report of trametinib-induced clearing of DD.^3^ Nevertheless, pan-RAF inhibition may have contributed by preventing paradoxical MAPK activation in wild-type keratinocytes, a phenomenon observed with selective BRAF inhibitors but not with pan-RAF inhibitors. Preclinical studies show that these agents inhibit RAF dimers across ARAF, BRAF, and CRAF isoforms, thereby maintaining balanced MAPK suppression without hyperactivation.^6^, ^7^, ^8^ By restoring physiological ERK signaling, trametinib, and potentially naporafenib, may counteract keratinocyte hyperactivation associated with SERCA2 dysfunction. Although trametinib was generally well tolerated in our patient, a transient episode of grade 3 hepatotoxicity occurred early during treatment. MEK inhibitors are known to cause a broad range of hepatic, gastrointestinal, and cardiac side effects, particularly with prolonged administration.^9^ While these toxicities are typically reversible, they underscore the need for close clinical and biochemical monitoring when such agents are considered in dermatologic indications.

## Conclusion

Together, these findings support MEK pathway inhibition as a rational therapeutic target for DD and highlight the need for mechanistic and clinical studies evaluating MAPK modulators in genodermatoses of keratinization. Long-term safety data in such indications remain absent, and cautious, individualized use is warranted until further evidence becomes available.

## Conflicts of interest

None disclosed.
